# The serum ANGPTL4 level and severe coronary artery calcification: from association to risk prediction using a nomogram

**DOI:** 10.3389/fcvm.2026.1784318

**Published:** 2026-05-07

**Authors:** Yan Sun, Mengchen Li, Dai Zhang, Yujing Cheng, Yu Liu, Jialong Niu, Hailong Ge, Xiaoli Liu, Hongya Han

**Affiliations:** Department of Cardiology, Beijing Anzhen Hospital, Capital Medical University, Beijing Institute of Heart Lung and Blood Vessel Disease, Beijing, China

**Keywords:** angiopoietin-like protein 4, atherosclerosis, coronary artery calcification, coronary artery disease, lipid metabolism

## Abstract

**Background:**

Coronary artery calcification (CAC) is an important indicator of atherosclerosis in the coronary arteries, the degree of which is closely associated with the risk for cardiovascular events. Angiopoietin-like protein 4 (ANGPTL4) plays a significant part in lipid metabolism and energy balance, and may also participate in the progression of CAC. This study was designed to examine the association between ANGPTL4 and CAC and to develop a nomogram to predict the risk of severe CAC.

**Methods:**

A total of 600 individuals who underwent multi-slice computed tomography (CT) of the coronary arteries and had available Agatston coronary artery calcification scores (CACS) at Beijing Anzhen Hospital were enrolled. Participants were divided into two groups: those with non-severe CAC (CACS ≤ 400) and those with severe CAC (CACS > 400). Determination of ANGPTL4 levels using an Enzyme-Linked Immunosorbent Assay (ELISA) kit. Identifying variables independently associated with severe CAC through least absolutely shrinkage selection operator (LASSO) regression analysis and logistic regression analysis.

**Results:**

Serum ANGPTL4 levels exhibited significantly higher concentrations in patients with severe CAC compared to those with non-severe CAC (*p* = 0.007). ANGPTL4 levels were positively associated with CAC severity, independent of other clinical risk factors. Age, smoking status, statin use, glycated hemoglobin A1c (HbA1c), serum phosphate, and ANGPTL4 were included in the predictive model for severe CAC. Receiver operating characteristic (ROC) curve analytics indicate that this model demonstrates acceptable discriminatory ability for severe CAC, with an area under the curve (AUC) of 0.708 [95% confidence interval (CI): 0.649–0.767].

**Conclusions:**

This study indicates that ANGPTL4 is independently associated with severe CAC, suggesting that ANGPTL4 could serve as a candidate biomarker and treatment target for vascular calcification.

## 1 Introduction

Coronary artery calcification (CAC) is a pathological condition distinguished by abnormal calcium and phosphate deposition within the coronary artery wall, serving as a valuable clinical indicator for underlying cholesterol-rich coronary atherosclerosis (1, 2). The coronary artery calcification score (CACS), which quantifies the extent of CAC, is commonly used to assess coronary atherosclerotic burden (3). Spotty calcification is often located within atherosclerotic lesions and contributes to an increased risk of plaque rupture (4, 5). As calcification progresses, vascular compliance decreases, and the complexity of interventional therapy increases (6). Although many studies have demonstrated a substantial association between the severity of CAC and increased cardiovascular risk, effective therapies for eliminating CAC remain lacking at present. Therefore, identifying novel targets for early prediction and preventive intervention is essential for the management of CAC.

Recently, increasing evidence has suggested the occurrence and progression of CAC is jointly driven by persistent dyslipidaemia. Several lipid parameters, including triglycerides (TG), apolipoprotein A-I (ApoA1), apolipoprotein B (ApoB), high-density lipoprotein cholesterol (HDL-C), and low-density lipoprotein cholesterol (LDL-C), have been widely recognized as being associated with CAC risk in the general population (7, 8). However, some individuals with CAC present with normal serum lipid levels. This observation suggests that abnormalities in lipid regulation may occur before measurable changes in serum lipids and lipoproteins become evident. Therefore, clarifying the association between lipid regulatory factors and the severity of CAC, and identifying novel indicators for the early identification of high-risk populations for CAC, holds significant clinical importance.

Angiopoietin-like proteins (ANGPTLs) constitute a family of secreted glycoproteins structurally analogous to angiopoietins, extensively implicated in the pathogenesis and progression of dyslipidaemia, diabetes mellitus, cancer, cerebral infarction, and cardiovascular diseases (CVDs) (9–11). Among these, angiopoietin-like protein 4 (ANGPTL4) plays a pivotal role in lipid metabolism and energy balance by inhibiting lipoprotein lipase (LPL) activity (12). Prior research indicates that ANGPTL4 overexpression reduces LPL activation and elevates circulating TG levels (9, 12). In addition, several population-based observational studies have demonstrated that ANGPTL4 loss-of-function variants are associated with lower TG levels, higher HDL-C levels, and reduced coronary artery disease (CAD) risk, supporting its potential utility as a biomarker for CAD (13–15). However, the direct involvement of ANGPTL4 in the pathogenesis of calcification remains unclear, necessitating further investigation regarding its function in vascular calcification. Notably, recent evidence indicates that ANGPTL4 is upregulated in severely calcified carotid plaques (16), suggesting that ANGPTL4 may play a role in the calcification pathogenesis. Therefore, it is necessary to conduct an in-depth assessment of the association between ANGPTL4 and CAC.

This study aims to determine whether ANGPTL4 is associated with CAC and to provide predictive insights that extend beyond traditional vascular risk factors. The pivotal aspect of this research is the development of a predictive model to assess the probability of developing severe CAC. The model holds promise for enhancing our understanding of the mechanism by which the lipid modulator ANGPTL4 contributes to the progression of CAC, and may aid in identifying individuals at high risk for severe and advanced atherosclerosis.

## 2 Methods

### 2.1 Study population

**Figure 1:**
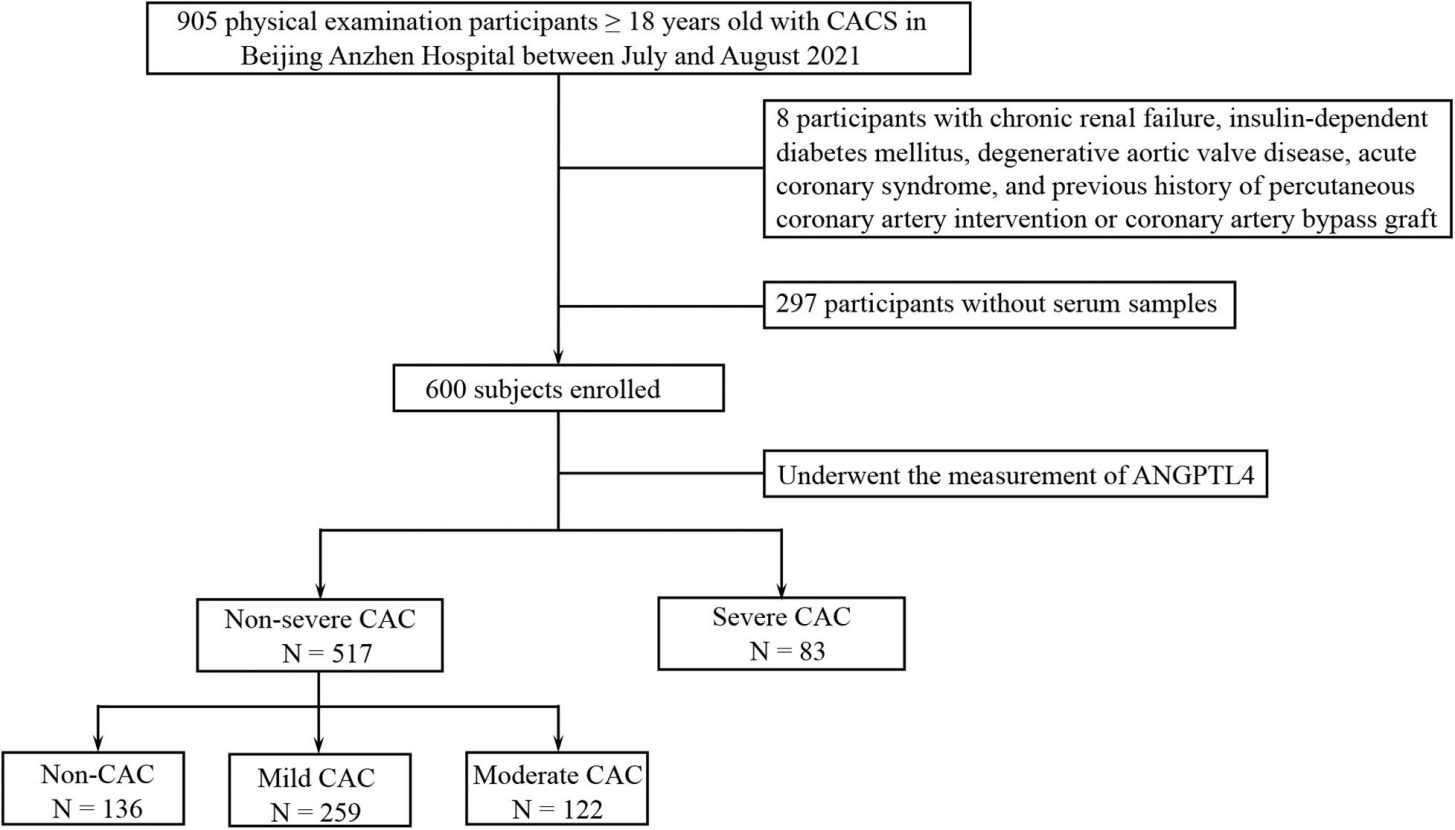
The flow chart for enrolling and grouping. CACS, coronary artery calcification score; ANGPTL4, angiopoietin-like protein 4; CAC, coronary artery calcification.

Between July and August 2021, 905 individuals undergoing routine physical examinations and CAC assessment at Beijing Anzhen Hospital were recruited. The present study was granted approval by the Ethics Committee of Beijing Anzhen Hospital (No: 2025011x), and obtained written informed consent from all participants. Criteria for inclusion were as follows: (1) age ≥ 18 years; (2) completion of a multi-slice spiral coronary computed tomography (CT) scan with CACS measured using the Agatston method; and (3) comprehensive clinical, laboratory, and imaging data. Individuals with chronic renal failure, acute coronary syndrome, insulin-dependent diabetes mellitus, degenerative aortic valve disease, history of percutaneous coronary intervention (PCI) or coronary artery bypass grafting (CABG) were excluded. After applying these criteria, 600 individuals were ultimately included in the analysis. A schematic diagram of the inclusion and exclusion process is shown in Figure 1.

### 2.2 Demographic and clinical information

Sociodemographic profile of each participant, including age, sex, body mass index (BMI), cardiovascular risk factors (arterial hypertension, hyperlipidemia, diabetes mellitus, smoking history, drinking history, and obesity), and treatment status (use of antihypertensive drugs, statins, and antidiabetic drugs), were recorded at the time of CT examination and blood sampling. Data on medication use were initially obtained through participant self-report and subsequently verified by the attending physicians. Biochemical parameters, including alanine aminotransferase (ALT), serum albumin (ALB), aspartate aminotransferase (AST), lipoprotein(a), total cholesterol (TC), triglycerides (TG), uric acid (UA), creatinine (Cr), fasting blood glucose (FBG), estimated glomerular filtration rate (eGFR), phosphate, high-sensitivity C-reactive protein (hsCRP), homocysteine, and glycated hemoglobin A1c (HbA1c), were measured within 24 h after admission.

### 2.3 Evaluation of CAC

Coronary computed tomography angiography (CCTA) was conducted using a dual-source CT scan system (Siemens SOMATOM Definition Flash, Berlin, Germany). The Agatston scoring method was used to calculate atherosclerotic plaques in the coronary arteries, with lesions defined as calcified plaques when CT attenuation values were ≥130 Hounsfield units (HU) and the lesion area was ≥0.5 mm^2^. Participants were classified into two groups according to CACS: the non-severe CAC cohort [CACS ≤ 400 AU (Agatston Units)] and the severe CAC cohort (CACS > 400 AU). The non-severe CAC cohort included individuals with non-CAC (CACS = 0), mild CAC (1 ≤ CACS < 100), and moderate CAC (100 ≤ CACS ≤ 400).

### 2.4 Determination of ANGPTL4 concentrations

Collect blood samples during CT scanning and centrifuge at 3,000 rpm for 10 min at 4 ℃ within two hours to separate serum from cellular components. Serum samples are frozen at −80 ℃ pending analysis. Serum ANGPTL4 levels were determined using an enzyme-linked immunosorbent assay (ELISA) kit (R&D Systems, Minneapolis, Minnesota, USA), with the procedure strictly adhering to the manufacturer's instructions. Triplicate measurements were performed for each sample. No significant cross-reactivity with other proteins was observed. To minimize inter-batch assay variability, all reagents used in the ANGPTL4 immunoassay were sourced from a single manufacturing batch. The intra-assay coefficients of variation (CVs) were 2.9%–5.3%, whereas the inter-assay CVs ranged from 5.8% to 8.6%.

### 2.5 Statistical analysis

Data analysis was conducted using SPSS software (version 27.0, IBM Corp., Armonk, NY, USA) and R software (version 4.5.0). Categorical variables are presented as numbers and percentages (*n*%). Continuously distributed variables are presented as mean ± standard deviation (SD) if normally distributed, or as median (25th and 75th percentiles) if non-normally distributed. Normality was assessed using the Kolmogorov–Smirnov test. Baseline characteristics were compared between the non-severe CAC and severe CAC groups using Student's t-test or the Mann–Whitney U test for continuous variables, and the chi-square test or Fisher's exact test for categorical variables, as appropriate. Pairwise correlations among study variables were assessed using Spearman rank correlation analysis and visualized by a correlation heatmap.

Before model construction, multicollinearity among candidate variables was evaluated using variance inflation factors (VIFs). Variables with VIF > 10 were sequentially removed until acceptable collinearity was achieved. Least absolute shrinkage and selection operator (LASSO) regression was then performed to identify potential predictors of severe CAC, and variables selected by LASSO were subsequently entered into logistic regression models to determine independent predictors. As a sensitivity analysis, Model 3 was further adjusted for TC and TG. A nomogram was generated based on the final multivariable logistic regression model for individualized prediction of severe CAC.

Model discrimination was assessed using receiver operating characteristic (ROC) curves and the corresponding area under the curve (AUC), with 95% confidence intervals (CIs). The incremental predictive value of adding ANGPTL4 to the baseline model was further evaluated using the category-free net reclassification improvement (NRI) and integrated discrimination improvement (IDI). Model calibration was assessed using calibration curves generated with the rms package, together with the calibration intercept, calibration slope, Brier score, and Spiegelhalter's *Z*-test. A two-sided *P* value < 0.05 was considered statistically significant.

## 3 Results

### 3.1 Characteristics of the study population

A summary of baseline characteristics for the study population (*n* = 600) is presented in Table 1. According to CAC severity, participants were categorized into a non-severe CAC cohort (*n* = 517) and a severe CAC cohort (*n* = 83). The non-severe CAC cohort was further stratified into three subgroups based on CACS: non-CAC (CACS = 0, *n* = 136), mild CAC (1 ≤ CACS < 100, *n* = 259), and moderate CAC (100 ≤ CACS < 400, *n* = 122).

**Table 1:** Clinical characteristics and ANGPTL4.

Variable	Non-severe CAC (CACS ≤ 400)				Severe CAC (CACS>400, *n* = 83)	*P*
	Non-CAC (*n* = 136)	Mild CAC (*n* = 259)	Moderate CAC (*n* = 122)	Total (*n* = 517)		
Age (years)	56.00 (53.00–58.00)	53.00 (48.00–57.00)	55.50 (51.00–58.00)	55.00 (50.00–57.00)	57.00 (53.00–58.00)	**<0** **.** **001**
Male sex, *n* (%)	136 (100.0)	259 (100.0)	122 (100.0)	517 (100.0)	83 (100.0)	-
Smoking, *n* (%)	96 (70.6)	196 (75.7)	83 (68.0)	375 (72.5)	71 (85.5)	**0** **.** **012**
Drinking, *n* (%)	111 (81.6)	216 (83.4)	102 (83.6)	429 (83.0)	74 (89.2)	0.156
Hypertension, *n* (%)	55 (40.4)	143 (55.2)	74 (60.7)	272 (52.6)	54 (65.1)	**0** **.** **035**
Diabetes, *n* (%)	21 (15.4)	69 (26.6)	42 (34.4)	132 (25.5)	31 (37.3)	**0** **.** **025**
Hyperlipidemia, *n* (%)	79 (58.1)	158 (61.0)	81 (66.4)	318 (61.5)	56 (67.5)	0.298
Diabetes medication, *n* (%)	15 (11.0)	52 (20.1)	29 (23.8)	96 (18.6)	26 (31.3)	**0** **.** **007**
Hypertension medication, *n* (%)	38 (27.9)	117 (45.2)	67 (54.9)	222 (42.9)	48 (57.8)	**0** **.** **011**
Statin, *n* (%)	22 (16.2)	54 (20.8)	40 (32.8)	116 (22.4)	35 (42.2)	**<0** **.** **001**
BMI (kg/m²)	26.44 (24.72–28.21)	26.73 (24.73–28.73)	27.02 (25.31–29.37)	26.68 (24.77–28.73)	25.76 (24.49–28.06)	0.104
ALB (g/L)	47.00 (44.60–49.15)	46.90 (43.40–49.20)	46.10 (43.60–48.20)	46.70 (44.00–49.10)	46.70 (43.10–49.30)	0.747
ALT (U/L)	19.00 (15.00–28.00)	20.00 (15.00–29.00)	20.00 (15.00–25.00)	20.00 (15.00–27.00)	20.00 (15.00–29.00)	0.601
AST (U/L)	22.00 (17.00–30.00)	22.00 (17.00–34.00)	20.00 (16.00–30.00)	21.00 (17.00–32.00)	25.00 (19.00–32.00)	0.065
Total cholesterol (mmol/L)	4.98 (4.20–5.76)	4.96 (4.12–5.67)	4.71 (3.93–5.44)	4.89 (4.12–5.67)	4.38 (3.60–5.41)	**0** **.** **004**
Triglyceride (mmol/L)	2.08 (1.50–3.27)	2.15 (1.57–3.09)	1.99 (1.32–3.02)	2.09 (1.48–3.07)	2.16 (1.56–3.25)	0.374
Lp(a) (nmol/L)	16.15 (6.20–36.60)	13.30 (7.10–42.00)	16.70 (7.90–48.00)	14.80 (7.10–40.30)	12.80 (6.30–34.40)	0.573
eGFR (mL/min)	104.98 (92.41–115.61)	104.15 (93.53–116.26)	104.47 (95.75–119.50)	104.28 (93.53–116.52)	103.67 (93.82–113.59)	0.626
Urea (mmol/L)	5.77 (5.09–6.52)	5.79 (4.94–6.77)	5.73 (5.05–6.97)	5.78 (5.02–6.77)	5.73 (4.91–6.81)	0.552
Uric acid (µmol/L)	351.00 (307.80–414.30)	345.50 (289.30–405.50)	342.00 (305.10–392.50)	345.90 (296.80–403.80)	348.30 (302.20–416.60)	0.439
Glucose (mmol/L)	5.50 (4.69–6.78)	5.87 (4.87–7.82)	5.88 (5.05–7.82)	5.72 (4.85–7.45)	5.98 (4.87–9.05)	0.100
PHOS (mmol/L)	1.12 (0.97–1.21)	1.11 (0.96–1.27)	1.09 (0.98–1.23)	1.10 (0.97–1.25)	1.17 (1.08–1.33)	**<0** **.** **001**
hsCRP (mg/L)	0.92 (0.53–1.42)	0.96 (0.58–1.73)	0.86 (0.51–1.46)	0.92 (0.55–1.63)	0.73 (0.40–1.42)	**0** **.** **023**
Homocysteine (µmol/L)	13.30 (11.60–16.10)	13.30 (11.30–15.80)	13.10 (11.70–15.10)	13.30 (11.50–15.50)	13.50 (11.60–15.80)	0.676
HbA1c (%)	5.70 (5.50–6.00)	5.80 (5.50–6.20)	5.80 (5.50–6.30)	5.80 (5.50–6.20)	6.00 (5.60–6.50)	**0** **.** **003**
CACS	0.00 (0.00–0.00)	20.00 (6.51–49.00)	181.70 (131.10–294.11)	18.88 (0.00–89.52)	801.87 (546.53–1,073.10)	**<0** **.** **001**
ANGPTL4	87.49 (70.12–108.48)	95.87 (76.97–120.80)	94.42 (77.53–126.12)	93.98 (75.37–118.79)	102.72 (81.05–137.99)	**0** **.** **007**

CAC, coronary artery calcification; BMI, body mass index; ALB, Albumin; ALT, alanine aminotransferase; AST, aspartate aminotransferase; Lp(a), lipoprotein(a); eGFR, estimated glomerular filtration rate; PHOS, phosphate; hsCRP, high-sensitivity C-reactive protein; HbA1c, glycated hemoglobin A1c; CACS, coronary artery calcification score; ANGPTL4, angiopoietin-like protein 4.

Bold values in the *P*-value column indicate statistical significance (*p* < 0.05).

**Figure 2:**
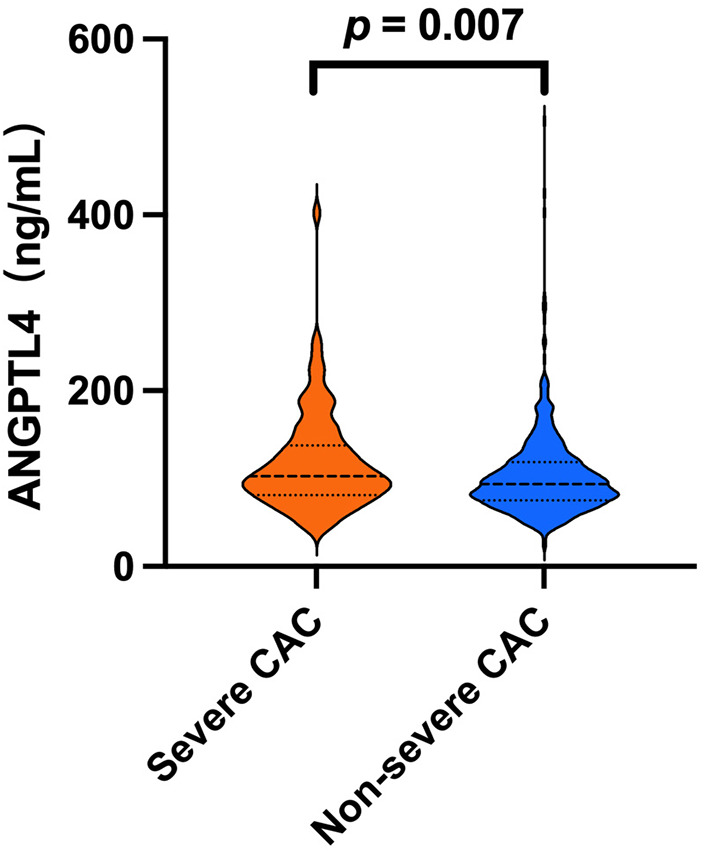
ANGPTL4 levels in the severe CAC and non-severe CAC cohorts. *P* values were calculated using the Mann–Whitney U test. ANGPTL4, angiopoietin-like protein 4; CAC, coronary artery calcification.

Analysis of clinical characteristics revealed that participants in the severe CAC cohort were older (*P* = 0.001) and exhibited higher prevalence rates of hypertension (*P* = 0.035), diabetes mellitus (*P* = 0.025), and smoking (*P* = 0.012). This group also had higher levels of ANGPTL4 (*P* = 0.007), HbA1c (*P* = 0.003), and serum phosphate (*P* = 0.001). Because the severe CAC cohort more frequently used statins (*P* < 0.001), total cholesterol levels in this group were lower than in the non-severe CAC cohort (*P* = 0.004). The two groups showed no significant differences in BMI, triglycerides, lipoprotein(a), urea, eGFR, uric acid, random glucose, or homocysteine levels. Serum ANGPTL4 levels in the two groups are shown in Figure 2.

**Figure 3:**
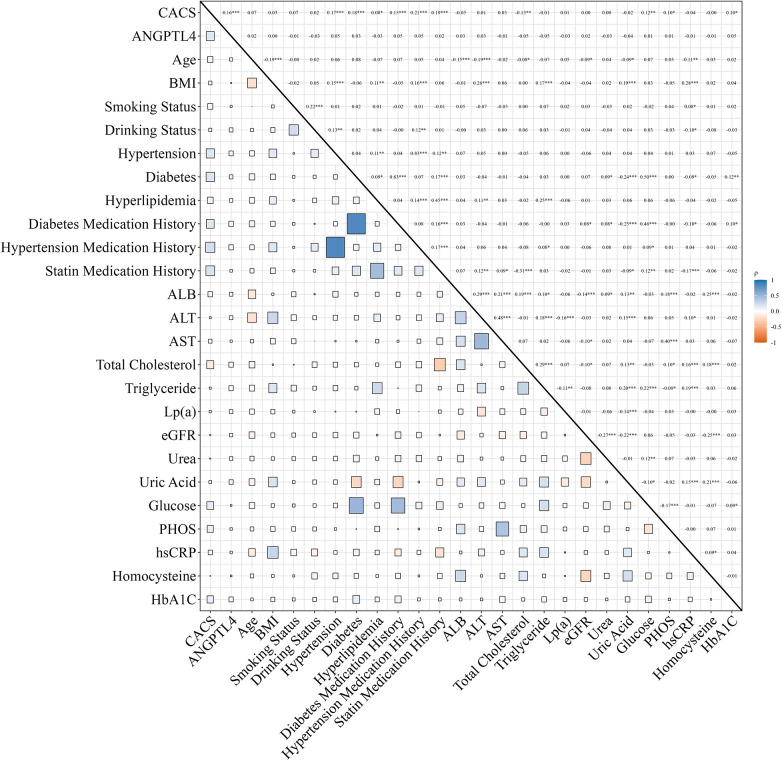
Spearman correlation analysis of variables showing statistically significant differences between the non-severe CAC cohort and the severe CAC cohort. Positive correlations are shown in blue and negative correlations in orange. Color intensity and square size represent the strength of the correlation. Statistical significance is indicated by asterisks (*P < 0.05, **P < 0.01, ***P < 0.001). CACS, coronary artery calcification score; ANGPTL4, angiopoietin-like protein 4; BMI, body mass index; ALB, albumin; ALT, alanine aminotransferase; AST, aspartate aminotransferase; Lp**(a)**, lipoprotein**(a)**; eGFR, estimated glomerular filtration rate; PHOS, phosphate; hsCRP, high-sensitivity C-reactive protein; HbA1c, glycated hemoglobin A1c.

As Figure 3 illustrates, Spearman correlation analysis was used to assess the relationships among ANGPTL4, CACS, and traditional cardiovascular risk factors. CACS was positively correlated with ANGPTL4 (*r* = 0.164, *P* < 0.05), diabetes mellitus (*r* = 0.176, *P* < 0.05), serum phosphate (*r* = 0.096, *P* < 0.05), hyperlipidemia (*r* = 0.083, *P* < 0.05), hypertension (*r* = 0.169, *P* < 0.05), and statin use (*r* = 0.193, *P* < 0.05).

### 3.2 LASSO regression and logistic regression analysis

**Figure 4:**
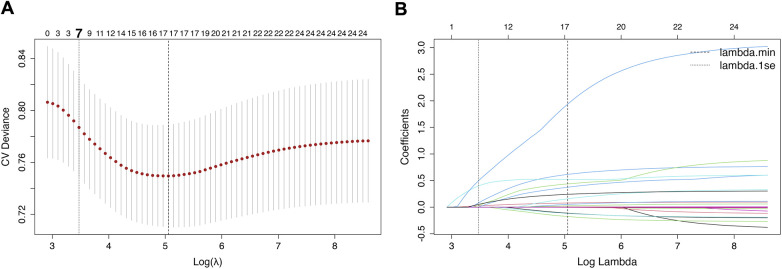
Identification of predictors using LASSO regression. **(A)** Cross-validation plot showing selection of the optimal penalty parameter (*λ* = 0.031013). **(B)** LASSO coefficient profile plot showing the seven variables retained longest as penalization increased. LASSO, least absolute shrinkage and selection operator.

As illustrated in Figure 4, predictors of severe CAC were identified using the LASSO regression method. Using the optimal penalty parameter (*λ*1se), seven candidate variables were identified from an initial set of 25 variables, including age, smoking, HbA1c, serum phosphate, statin use, antidiabetic drug use, and ANGPTL4.

As shown in Table 2, univariate logistic regression analysis demonstrated that older age [odds ratio (OR) = 1.100, 95% confidence interval (CI): 1.038–1.165, *P* = 0.001], smoking (OR = 2.240, 95% CI: 1.180–4.255, *P* = 0.014), statin use (OR = 2.521, 95% CI: 1.556–4.082, *P* < 0.001), antidiabetic drug use (OR = 2.000, 95% CI: 1.196–3.345, *P* = 0.008), HbA1c (OR = 1.341, 95% CI: 1.071–1.679, *P* = 0.011), serum phosphate (OR = 4.908, 95% CI: 1.844–13.063, *P* = 0.001), and higher ANGPTL4 levels (OR = 1.005, 95% CI: 1.001–1.009, *P* = 0.011) were positively associated with severe CAC (Model 1). After adjustment for age, smoking (OR = 2.274, 95% CI: 1.192–4.337, *P* = 0.013), statin use (OR = 2.479, 95% CI: 1.523–4.037, *P* < 0.001), antidiabetic drug use (OR = 1.886, 95% CI: 1.122–3.171, *P* = 0.017), HbA1c (OR = 1.369, 95% CI: 1.086–1.726, *P* = 0.008), serum phosphate (OR = 4.653, 95% CI: 1.734–12.486, *P* = 0.002), and ANGPTL4 levels (OR = 1.005, 95% CI: 1.001–1.009, *P* = 0.016) remained significantly associated with severe CAC (Model 2). In the fully adjusted model (Model 3), which included age, smoking status, statin use, antidiabetic drug use, HbA1c, serum phosphate, and ANGPTL4, several variables remained independently associated with severe CAC. Importantly, ANGPTL4 continued to show an independent association with severe CAC after adjustment for all covariates (OR = 1.005, 95% CI: 1.001–1.009, *P* = 0.014).

**Table 2:** Univariate and multivariate analysis of factors associated with severe CAC.

Variable	Model 1		Model 2		Model 3	
	OR (95% CI)	*P*	OR (95% CI)	*P*	OR (95% CI)	*P*
Age	1.100 (1.038–1.165)	0.001	-	-	1.097 (1.033–1.166)	0.003
Smoking	2.240 (1.180–4.255)	0.014	2.274 (1.192–4.337)	0.013	2.319 (1.192–4.511)	0.013
Statin Use	2.521 (1.556–4.082)	<0.001	2.479 (1.523–4.037)	<0.001	2.364 (1.416–3.948)	0.001
Anti-diabetes Drug Use	2.000 (1.196–3.345)	0.008	1.886 (1.122–3.171)	0.017	1.585 (0.913–2.751)	0.102
HbA1c	1.341 (1.071–1.679)	0.011	1.369 (1.086–1.726)	0.008	1.305 (1.021–1.668)	0.033
Phosphate	4.908 (1.844–13.063)	0.001	4.653 (1.734–12.486)	0.002	5.073 (1.785–14.420)	0.002
ANGPTL4	1.005 (1.001–1.009)	0.011	1.005 (1.001–1.009)	0.016	1.005 (1.001–1.009)	0.014

OR, odds ratio; CI, confidence interval; CAC, coronary artery calcification; HbA1c, glycated hemoglobin A1c; ANGPTL4, angiopoietin-like protein 4.

Model 1: Univariate logistic regression.

Model 2: Logistic regression after adjusted age.

Model 3: adjusted for age, smoking, statin utilization, anti-diabetes drug utilization, HbA1c and phosphate.

To further address potential confounding by lipid-related factors, we performed a sensitivity analysis with additional adjustment for TC and TG. After this adjustment, the association between ANGPTL4 and severe CAC remained statistically significant in Model 3 (OR = 1.005, 95% CI: 1.001–1.009, *P* = 0.022), indicating the robustness of the observed association (Supplementary Table S1).

### 3.3 ROC and nomogram of ANGPTL4 prediction model

**Figure 5:**
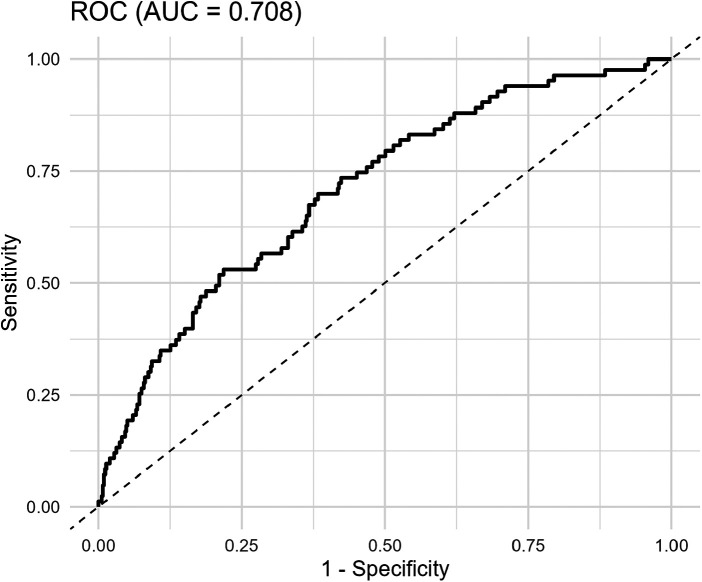
ROC curve of ANGPTL4 prediction model. ROC curve, receiver operating characteristic curve; AUC, area under the curve.

Based on multivariate logistic regression analysis results, a predictive model incorporating age, smoking status, statin use, HbA1c, serum phosphate, and ANGPTL4 was constructed to assess the risk of severe CAC. Performance of the model has been evaluated by means of ROC curve, as illustrated in Figure 5. The AUC was 0.708 (95% CI: 0.649–0.767), indicating that the model has good discriminatory capability.

**Figure 6:**
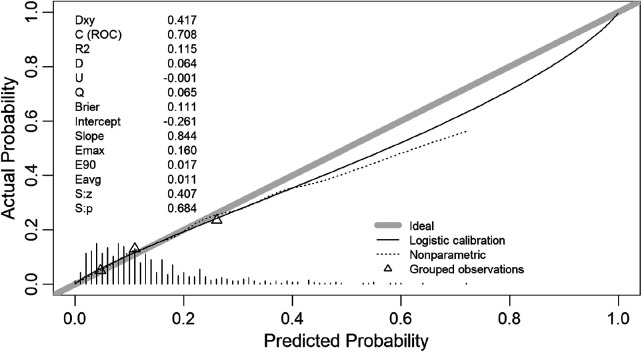
Calibration curve of ANGPTL4 prediction model.

To evaluate model accuracy, calibration analysis was performed. The calibration curve demonstrates a high degree of concordance between predicted and observed probabilities of severe CAC, with no evident overestimation or underestimation across the range of predicted risks (Figure 6). Spiegelhalter's *Z*-test showed no evidence of systematic miscalibration (Z = 0.407, *P* = 0.684), supporting satisfactory model calibration. In incremental analyses, the addition of ANGPTL4 to the baseline risk model resulted in a significant improvement in the continuous NRI of 0.2476 (95% CI: 0.0342–0.4938, *P* = 0.030), whereas the IDI showed a positive but non-significant change of 0.0070 (95% CI: −0.0028 to 0.0178, *P* = 0.178) (Supplementary Table S2).

**Figure 7:**
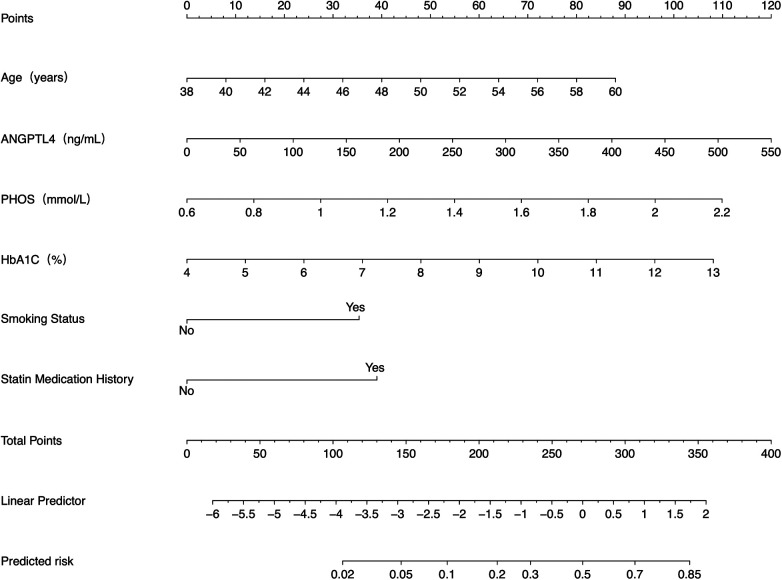
Nomogram to predict the risk of severe CAC occurrence. ANGPTL4, angiopoietin-like protein 4; PHOS, phosphate; HbA1c, glycated hemoglobin A1c.

In addition, a nomogram was developed based on the final multivariate logistic regression model. Variables included in the nomogram were first selected by LASSO regularization and then confirmed in the fully adjusted model, ensuring that only robust predictors of severe CAC were incorporated (Figure 7).

## 4 Discussion

Our study yielded two main findings. First, serum ANGPTL4 levels were independently associated with severe CAC. Second, a predictive model for severe CAC was developed and validated using readily available clinical factors, including age, smoking status, statin use, HbA1c, serum phosphate, and serum ANGPTL4 levels. This evidence suggests that ANGPTL4 plays a significant role in the pathogenesis of severe CAC and may provide novel insights into the development of coronary atherosclerosis.

CAC serves as a marker of the overall burden of atherosclerosis and is closely associated with adverse vascular events and all-cause mortality (17, 18). Increasing evidence indicates that factors related to lipid metabolism contribute substantially to the development of CAC and atherosclerosis. ANGPTLs, which are extensively expressed in the liver, vascular system, and hematopoietic tissues, are key regulators of lipid metabolism (12). Among them, ANGPTL4 is a polyfunctional protein whose mechanisms influencing calcification and atherosclerosis may extend beyond its role in regulating circulating lipid levels (19). In atherosclerotic mouse models, ANGPTL4 has been reported to exert anti-atherogenic effects by reducing plaque size and vascular inflammation (20, 21). Consistent clinical observations indicate that patients with higher plasma ANGPTL4 levels following acute myocardial infarction (AMI) exhibit a lower incidence of subsequent cardiovascular events compared to those who have low levels (20). In contrast, several clinical studies indicate that ANGPTL4 is positively correlated with cardiovascular risk. A longitudinal community-based cohort comprising 1,163 participants with a median follow-up of 10.46 years revealed that participants with the highest tertile of plasma ANGPTL4 levels exhibited the lowest survival rates. Furthermore, ANGPTL4 independently predicted cardiovascular or cancer-related mortality even after comprehensive adjustment for potential confounders, including age, sex, smoking, body mass index, hypertension, diabetes mellitus, and renal function (22). In the LURIC and getABI cohorts, higher ANGPTL4 levels were associated with increased cardiovascular mortality and fatal myocardial infarction (9). Furthermore, Silbernagel et al. (23) reported that ANGPTL4 levels were positively correlated with both the progression of CAC (assessed by Agatston score) and the incidence of cardiovascular events. In line with these findings, after adjusting for conventional coronary heart disease risk factors, this study further confirms a positive and independent association between ANGPTL4 and severe CAC. Taken together, current evidence is limited to establishing an association between ANGPTL4 and atherosclerosis, precluding definitive causal conclusions. Moreover, heterogeneity in sample sizes and study population characteristics has contributed to inconsistent findings. Accordingly, large-scale, multi-center, prospective studies are warranted to confirm these observations.

Given the exclusion of patients with prior PCI or CABG, the present study focuses on the pathophysiological process of early-to-mid stages of CAD, rather than the most advanced disease stage. This is particularly relevant given the hypothesis that ANGPTL4 acts as a regulator of plaque homeostasis, facilitating calcific stabilization during atherogenesis. However, this homeostatic mechanism may already be disrupted in more advanced lesions. In addition, patients requiring revascularization often exhibit more severe ischemia, myocardial injury, and systemic inflammation, which could confound circulating ANGPTL4 levels and obscure its relationship with local plaque biology. This may partly account for the dual or context-dependent role of ANGPTL4 observed in CAD patients. Therefore, our exclusion criteria may enable an unconfounded assessment of the interplay between ANGPTL4 and coronary atherosclerotic progression. Future studies will include subgroup analyses of advanced CAD patients without revascularization and employ advanced imaging techniques to characterize plaque composition, thereby clarifying the role of ANGPTL4 in advanced stage of CAD.

To elucidate the precise biological mechanisms linking ANGPTL4 to vascular calcification, accumulating evidence indicates that ANGPTL4 exerts dual regulatory functions in a tissue- and microenvironment-dependent manner (24, 25). Systemically, ANGPTL4 promotes vascular calcification by inhibiting LPL activity, raising plasma triglycerides and indirectly promoting an atherogenic lipid environment. Locally, ANGPTL4 functions as a pathologically inducible regulator in vascular smooth muscle cells (VSMCs), serving as a pivotal nexus between lipid metabolism disorders and osteogenic transformation. Chellan et al. confirmed that enzyme-modified low-density lipoprotein (E-LDL) could significantly increase the level of ANGPTL4 in VSMCs and drive osteogenic transdifferentiation (26). Conversely, in macrophages, ANGPTL4 acts as a protective feedback regulator by inhibiting foam cell formation and attenuating plaque inflammation, thereby contributing to lesion stability and exerting indirect anti-calcification effects (21). Collectively, these data indicate that the role of ANGPTL4 in vascular calcification and atherosclerosis remains complex and controversial. We may speculate that ANGPTL4 may initially serve a protective role in intraplaque cells by mitigating lipotoxicity during early atherogenesis. However, sustained upregulation under pathological conditions could drive a maladaptive shift toward promoting vascular inflammation, VSMC migration, and osteogenic transdifferentiation, thereby accelerating atherosclerosis progression.

There is a complex interaction among CAC, lipid metabolism, and atherosclerosis progression. Statin therapy is the cornerstone of lipid management and cardiovascular risk reduction; however, accumulating evidence suggests that statin use may be associated with increased CAC (27). Henein et al. (28) demonstrated that long-term statin therapy, particularly at high doses, accelerates CAC. Similarly, a study involving 3,483 participants revealed that the progression of CAC among statin users remained 31% higher, even after adjustment for cardiovascular risk parameters (29). Our findings are consistent with these observations, as statin use is significantly correlated with an elevated risk of developing severe CAC. Although this effect may appear unfavorable, increased calcification has been linked to greater plaque stability and fewer adverse cardiovascular events, likely through reduction of the lipid-rich core within atherosclerotic plaques (30). Numerous studies have sought to clarify the underlying mechanisms of this effect (31). On one hand, statin therapy exerts anti-inflammatory and lipid-regulating effects to stabilize the plaque environment. On the other hand, it actively remodels the plaque microstructure by promoting the osteogenic differentiation of VSMCs, modulating the activity of calcification inhibitors, and facilitating the fusion of microcalcifications into dense, macro-calcifications. Consequently, this shifts the plaque phenotype from a vulnerable, high-lipid, microcalcification state to a stable, low-lipid, macro-calcification configuration. However, further research is required to validate these findings and elucidate their underlying mechanisms.

The study also identified additional factors associated with CAC, including age, HbA1c, smoking and serum phosphate levels. Advanced age is a recognized risk factor for CAC (32). Regression analysis revealed that advanced age was significantly associated with an increased risk of severe CAC, consistent with findings from previous studies. Smoking remains a major global health concern and contributes substantially to cardiovascular morbidity and mortality. Most existing research has confirmed the association between smoking and CAC from multiple perspectives (33, 34). Consistent with these findings, the current study demonstrates that smoking is a significant risk factor for severe CAC. HbA1c, a standardized marker of long-term glycemic control, has been shown to be positively associated with CACS in several studies (35, 36). The current results further support the relationship between elevated HbA1c levels and severe CAC. Serum phosphate levels are associated with atherosclerosis in various pathological conditions, particularly in patients with chronic kidney disease (CKD) and severe hyperphosphatemia (37). However, the contribution of phosphate to CAC has primarily been investigated in the context of hyperphosphatemia, and findings regarding its association with CAC in the general population remain inconsistent. Gronhøj et al. (38) reported that serum phosphate levels were not associated with the prevalence of CAC. In contrast, Campos-Obando et al. (39) found significant associations between serum phosphate and CAC even after excluding patients with hyperphosphatemia, CKD, and established cardiovascular disease. Similarly, the present analysis indicates that serum phosphate levels are associated with CAC in male Chinese subjects with normal renal function, and this association occurs even when serum phosphate remains within the normal range. Nevertheless, the underlying mechanisms linking serum phosphate levels to the incidence and mortality of cardiovascular disease remain unclear.

Unlike conventional risk stratification, nomograms can offer individualized risk quantification by integrating weighted contributions of multiple predictors, such as clinical parameters, serum biomarkers, and imaging features, into a visualized scoring system. In the management of cardiovascular diseases, nomograms have demonstrated broad applicability across multiple clinical scenarios, including risk screening and early warning, prognostic assessment following PCI, guidance for individualized treatment plans, and clinical validation of emerging biomarkers. For instance, Shen *et al*. (40) constructed a nomogram incorporating the novel cardiac biomarker cardiac myosin-binding protein-C (cMyBP-C) alongside conventional risk factors to predict the incidence of cardiovascular disease, achieving good predictive performance (AUC = 0.816 in the training cohort; 0.774 in the validation cohort). Similarly, among elderly patients undergoing PCI, nomograms have demonstrated robust utility in predicting the risk of adverse procedural outcomes (41). In the present study, we demonstrate that a nomogram integrating ANGPTL4 with traditional risk factors effectively predicts the occurrence of severe CAC. Looking forward, it is plausible to hypothesize that future nomogram-based risk models incorporating a broader panel of novel biomarkers and advanced imaging parameters could further aid physicians in identifying high-risk patients requiring more intensive management, such as aggressive antiplatelet therapy or frequent imaging follow-up. However, before nomograms can be widely implemented in clinical settings, rigorous external validation, comprehensive performance evaluation, and due consideration of population-specific characteristics are required.

This study has several limitations that require clarification. First, cross-sectional study designs cannot establish a causal relationship between ANGPTL4 and the severity of CAC, nor can they assess the long-term impact of ANGPTL4 on the progression of CAC. Second, the single-center design of this study and its focus on a solely Chinese male population restrict the broader applicability of the results. Given the well-established sex-specific disparities in CAD, where the peak incidence in women typically occurs 7–10 years later than in men, predominantly after menopause (>55 years), the average age of our cohort (55 years) was associated with a relatively low disease prevalence in women. To ensure a balanced comparison between groups, we therefore restricted our initial analysis to male participants. Furthermore, the pathophysiology of CAD and CAC in women is characterized by a more complex interaction of sex-specific factors, such as hormonal fluctuations and pregnancy-related history. Consequently, multi-center studies with larger, mixed-gender populations are essential to validate these findings. Third, while the nomogram model developed in this study exhibits promising predictive performance for severe CAC, it requires external validation. Future work will involve rigorous validation in large-scale, multi-center, and geographically diverse populations to confirm the robustness and generalizability of this prediction tool. Fourth, CACS itself has inherent limitations as a measure of CAC. For example, individuals who have previously undergone PCI, particularly coronary stent implantation, are not eligible for accurate CACS assessment.

## 5 Conclusions

In summary, the present study demonstrates that elevated ANGPTL4 concentrations are independently associated with severe CAC, suggesting that ANGPTL4 could play a significant role in the progression of atherosclerosis. Findings indicate that targeting ANGPTL4 may represent an effective strategy for the prevention and treatment of CAD.

## Data Availability

The datasets presented in this article are not readily available because they contain potentially sensitive patient information and are subject to patient privacy restrictions. Requests to access the datasets should be directed to the corresponding author.
