# Supplementary material for: The serum ANGPTL4 level and severe coronary artery calcification: from association to risk prediction using a nomogram
Source: Front Cardiovasc Med. 2026 May 7;13:1784318. doi: 10.3389/fcvm.2026.1784318 (PMC13190530; doi:10.3389/fcvm.2026.1784318)
Supplement: Supplementary file 1 [file Datasheet1.docx]

**Supplementary Materials**

The Serum ANGPTL4 Level and Severe Coronary Artery Calcification: From Association to Risk Prediction Using a Nomogram

**Table S1. Sensitivity analysis with additional adjustment for total cholesterol and triglycerides.**

| Variable | Model 1 | | Model 2 | | Model 3 | |
| --- | --- | --- | --- | --- | --- | --- |
|  | OR (95% CI) | *P* | OR (95% CI) | *P* | OR (95% CI) | *P* |
| Age | 1.100 (1.038–1.165) | 0.001 | - | - | 1.098 (1.033–1.168) | 0.003 |
| Smoking | 2.240 (1.180–4.255) | 0.014 | 2.274 (1.192–4.337) | 0.013 | 2.299 (1.176–4.494) | 0.015 |
| Statin Use | 2.521 (1.556–4.082) | <0.001 | 2.479 (1.523–4.037) | <0.001 | 1.932 (1.122–3.327) | 0.017 |
| Anti-diabetes Drug Use | 2.000 (1.196–3.345) | 0.008 | 1.886 (1.122–3.171) | 0.017 | 1.583 (0.910–2.752) | 0.104 |
| HbA1c | 1.341 (1.071–1.679) | 0.011 | 1.369 (1.086–1.726) | 0.008 | 1.312 (1.027–1.677) | 0.03 |
| Phosphate | 4.908 (1.844–13.063) | 0.001 | 4.653 (1.734–12.486) | 0.002 | 6.645 (2.262–19.521) | <0.001 |
| ANGPTL4 | 1.005 (1.001–1.009) | 0.011 | 1.005 (1.001–1.009) | 0.016 | 1.005 (1.001–1.009) | 0.022 |
| Total Cholesterol | 0.774 (0.625–0.958) | 0.019 | 0.797 (0.644–0.987) | 0.037 | 0.764 (0.598–0.977) | 0.032 |
| Triglycerides | 1.047 (0.933–1.175) | 0.436 | 1.069 (0.951–1.203) | 0.264 | 1.143 (0.992–1.317) | 0.064 |

OR, odds ratio; CI, confidence interval; CAC, coronary artery calcification; HbA1c, glycated hemoglobin A1c; ANGPTL4, angiopoietin‐like protein 4.

Model 1: Univariate logistic regression.

Model 2: Logistic regression after adjusted age.

Model 3: adjusted for age, smoking, statin utilization, anti-diabetes drug utilization, HbA1c, total cholesterol, triglycerides and phosphate.

**Table S2. Incremental predictive performance of adding ANGPTL4 to the baseline risk model: NRI and IDI analyses.**

| Model | NRI (95% CI) | *P* | IDI (95% CI) | *P* |
| --- | --- | --- | --- | --- |
| Baseline risk model | Ref. |  | Ref. |  |
| +ANGPTL4 | 0.248 (0.034-0.494) | 0.030 | 0.007 (-0.003-0.018) | 0.178 |

NRI, net reclassification improvement; IDI, integrated discrimination improvement; CI, confidence interval; ANGPTL4, angiopoietin‐like protein 4.

Baseline risk model included age, smoking, statin utilization, HbA1c and phosphate. +ANGPTL4 indicates the baseline model additionally including ANGPTL4.
